# Quantifying the impact of tumor size and motion on 4DCT‐4DCBCT image registration accuracy using machine learning and statistical analysis

**DOI:** 10.1002/acm2.70503

**Published:** 2026-02-10

**Authors:** Qiaoyan Jing, Shuyu Lin, Binyun Huang, Tingjun Luo, Xianya Li, Weiming Zhang, Shaohan Sun

**Affiliations:** ^1^ Department of Radiation Oncology Wuming Hospital of Guangxi Medical University Nanning People's Republic of China; ^2^ Department of Radiation Oncology Guangxi Medical University Cancer Hospital Nanning People's Republic of China

**Keywords:** 4D CBCT, 4D CT, Dice similarity coefficient, image registration, random forest analysis

## Abstract

**Purpose:**

This study systematically quantifies the effects of five variables—respiratory cycle, tumor size, and motion amplitudes in the superior‐inferior (SI), anterior‐posterior (AP), and left‐right (LR) directions—on the registration accuracy between four‐dimensional computed tomography (4D CT) and four‐dimensional cone‐beam CT (4D CBCT) images, thereby providing a theoretical basis for optimizing registration strategies in image‐guided radiotherapy (IGRT).

**Materials and methods:**

A CIRS 008A dynamic phantom fitted with 1 and 3 cm tumor inserts was utilized to simulate various respiratory motion scenarios by manipulating respiratory cycles (*T* = 0, 2, 4, and 8 s) and three‐dimensional motion amplitudes (SI, AP, and LR ranging from 0 to 15 mm, with AP and LR limited to 0, 1, and 5 mm). Corresponding four‐dimensional images were acquired using a GE Discovery RT CT simulator and a Varian VitalBeam linear accelerator. Rigid registration between the 4D CT and 4D CBCT images was subsequently performed using the Varian imaging system, with registration quality quantitatively assessed via the Dice similarity coefficient (DSC). Furthermore, a Random Forest regression model was employed to determine the relative importance of each factor, and multifactor analysis of variance (ANOVA) was conducted to verify statistical significance.

**Results:**

The Random Forest analysis indicated that, for the overall registration average intensity projection, the factors were ranked in order of importance as follows: tumor size (0.509), SI motion (0.315), respiratory cycle (0.094), LR motion (0.055), and AP motion (0.028). In the maximum intensity projection, tumor size (0.722) was found to have a particularly significant impact. The multifactor ANOVA further supported these findings, demonstrating that tumor size (*p* < 0.001) and SI motion (*p* < 0.001) have a highly significant influence on registration quality, whereas the respiratory cycle and AP/LR motions did not reach statistical significance (*p* > 0.05). Notably, when the tumor size was small (1 cm) and accompanied by considerable SI motion (>10 mm), registration accuracy markedly deteriorated, with the greatest variability observed under these conditions.

**Conclusion:**

This study demonstrated that the registration quality between 4D CT and 4D CBCT images was significantly influenced by both tumor size and the amplitude of motion in the SI direction.

## 1 INTRODUCTION

Radiation therapy is a primary tumor treatment modality whose effectiveness depends critically on precise target delineation and accurate dose delivery. Advances in Image‐Guided Radiation Therapy (IGRT)^1^ have established four‐dimensional computed tomography (4D CT) and four‐dimensional cone‐beam computed tomography (4D CBCT) as essential imaging tools in contemporary radiotherapy systems. These modalities effectively quantify and compensate for target displacement and deformation resulting from respiratory motion^2^, ^3^ thereby supporting the delivery of precise radiotherapy. Nonetheless, achieving accurate registration between 4D CT and 4D CBCT images remains challenging, particularly given the complexities of organ motion and deformation.^4^

The accuracy of 4D image registration is influenced by various physical and physiological factors. Previous investigations have demonstrated that respiratory motion patterns, tumor characteristics, and imaging parameters significantly affect registration accuracy.^5^ In particular, tumor size is a critical factor; smaller tumors frequently increase registration uncertainty due to partial volume effects and diminished image contrast.^6^ Additionally, the amplitude and directional attributes of respiratory motion—most notably the superior‐inferior (SI) movement driven predominantly by diaphragmatic activity—exhibit the greatest range of motion, which poses substantial challenges to registration algorithms.^7^ However, systematic studies that quantify the relative importance of these factors, as well as their interactions and impacts on registration quality, remain limited.^8^

Existing research into registration quality assessment has generally focused on single‐factor analyses or validations within specific clinical contexts, rather than providing a comprehensive evaluation of the various contributing factors.^9^, ^10^ Traditional statistical approaches often struggle to address the complex nonlinear relationships among multiple factors, whereas machine learning techniques, such as random forest regression, can effectively quantify the importance of individual features^11^ thereby offering new insights into multifactor systems. Integrating this approach with traditional variance analysis enables simultaneous evaluation of factor importance and statistical significance^12^ strengthening clinical decision‐making.

In this study, a CIRS 008A motion phantom was utilized to simulate realistic respiratory motion, with four‐dimensional images acquired using a GE Discovery RT CT simulator and a Varian VitalBeam linear accelerator imaging system. Systematic machine learning and statistical analyses were employed to quantitatively evaluate the impacts of respiratory phase, tumor size, and multidirectional motion amplitudes on the registration quality between 4D CT and 4D CBCT images. The Dice similarity coefficient (DSC), widely recognized clinical metrics, was used to quantify registration quality, including DSC values for each respiratory phase as well as those derived from average intensity projection (AIP) and maximum intensity projection (MIP) images. This study combined random forest feature importance with multifactor variance analysis to assess factor contributions and verify significance. The findings may optimize 4D image registration, tailor personalized strategies for various tumor characteristics and motion patterns, and support clinical plan adaptation and motion management.

## 2 MATERIALS AND METHODS

### 2.1 Motion simulation model and motion trajectory design

**FIGURE 1:**
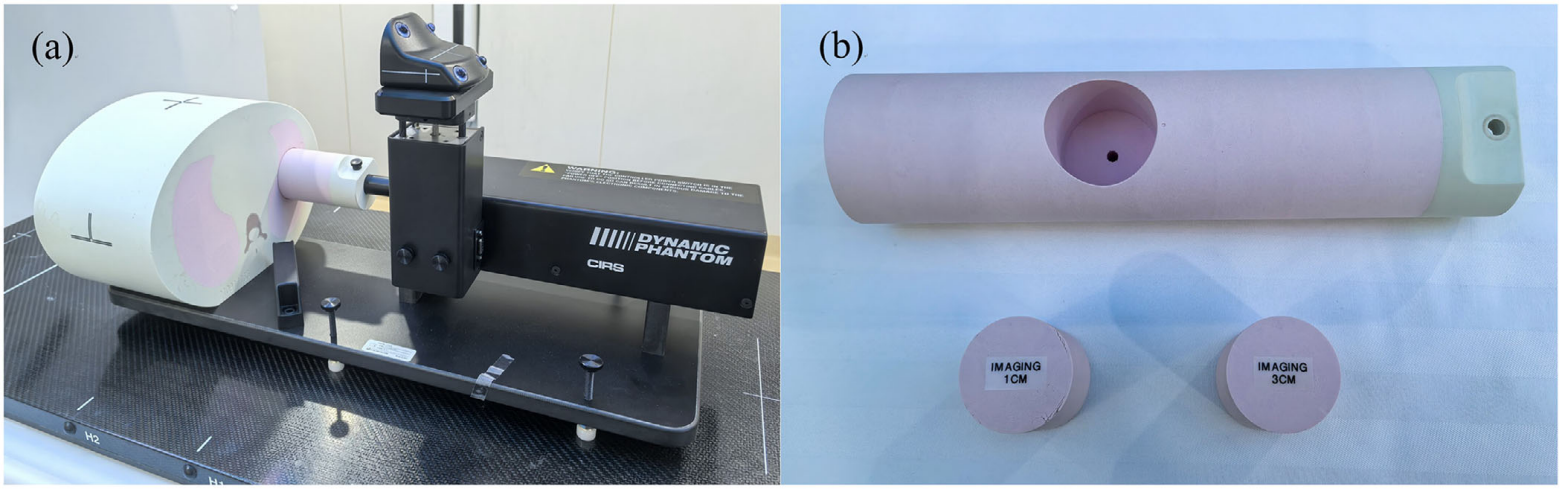
Photograph of the model 008A dynamic phantom: (a) composed of the chest module and movable platform; (b) featuring the lung‐equivalent rod along with 1 and 3 cm solid tumor plugs.

In this study, we employed a Model 008A dynamic phantom manufactured by CIRS Inc. (Norfolk, VA, USA). This device comprises a chest tissue‐equivalent module and a mobile platform with programmable motor control (see Figure 1a), which accurately replicates the relative motion between a tumor and its surrounding tissues during human respiration. The phantom's positional accuracy is reported by the manufacturer as ± 0.1 mm. The platform accommodates tumor inserts of various sizes; for this investigation, spherical inserts with diameters of 1 and 3 cm were selected (see Figure 1b).

**FIGURE 2:**
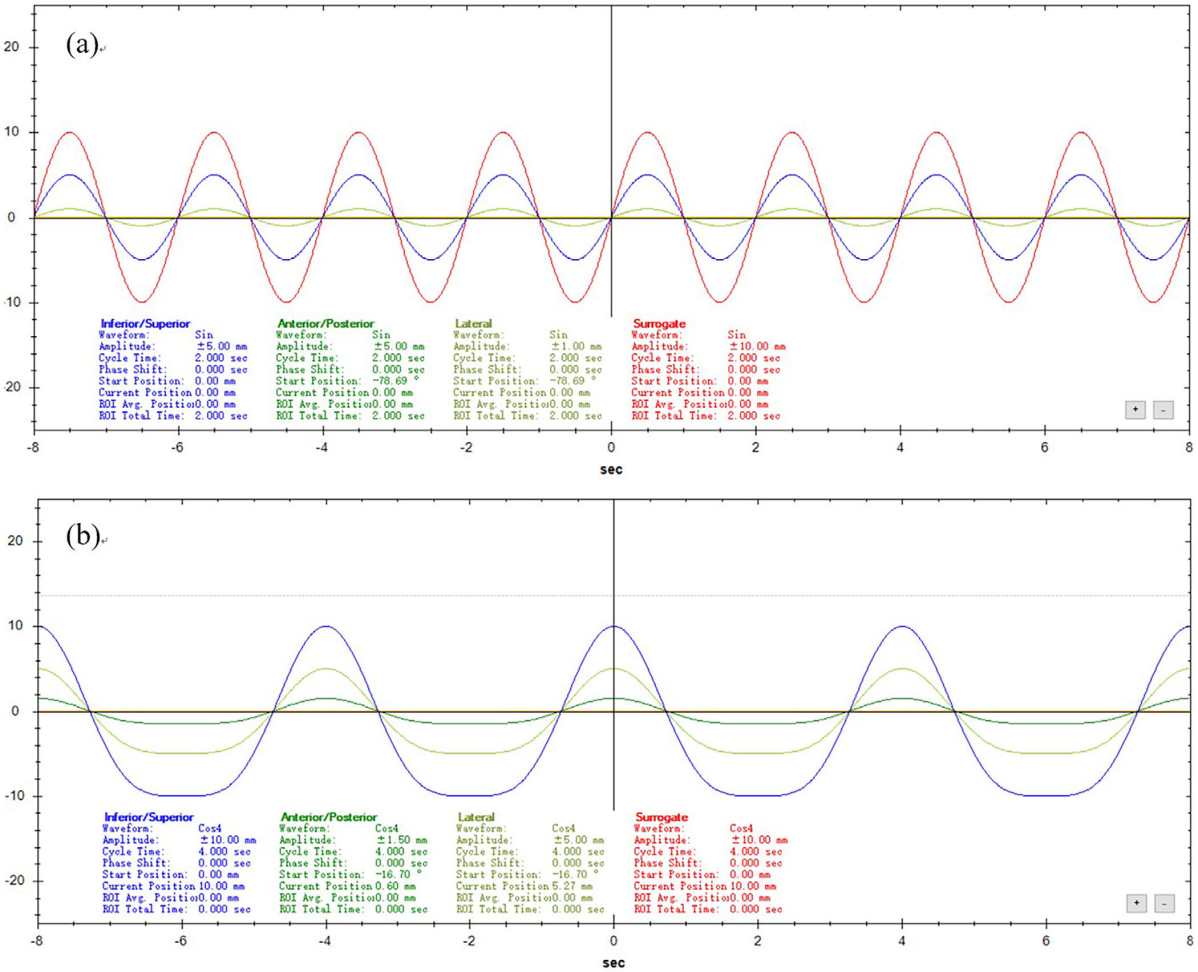
Experimental design: motion waveforms. (a) Motion waveform with a period of 2 seconds; (b) motion waveforms with periods of 4 seconds and 8 seconds.

Dedicated phantom software was utilized to design and control the tumor motion trajectory precisely and to develop a multivariate experimental scheme for evaluating the effects of various factors on the accuracy of 4D image registration. The three‐dimensional experimental motion trajectories—SI, anterior‐posterior (AP), and left‐right (LR)—were rigorously maintained in accordance with the following design principles: (1) Motion waveforms: For a 2‐second period, a COS waveform was applied in all directions (see Figure 2a), simulating the respiratory waveform during rapid breathing; for periods of 4 and 8 seconds, a COS4 waveform was used (see Figure 2b) to replicate typical respiratory patterns, as commonly employed in previous studies.^13^, ^14^ (2) Breathing cycle: To mimic the diverse respiratory patterns observed in patients, three distinct breathing cycles were established: 2 seconds (30 breaths per minute, representing rapid breathing), 4 seconds (15 breaths per minute, representing normal breathing), and 8 seconds (7.5 breaths per minute, representing slow breathing). (3) Motion amplitude: These amplitude values were selected to reflect clinically observed ranges of lung tumor motion.^15^ Four amplitude values were designated along the primary motion direction (SI): 0, 5, 10, and 15 mm, encompassing scenarios from static to large‐amplitude motion. In the secondary motion directions (AP and LR), where motion amplitudes are typically lower, three amplitude values were set: 0, 1, and 5 mm. The experiment acquired 52 four‐dimensional image sets featuring a range of tumor sizes, respiratory cycles, and three‐dimensional motion amplitudes, as well as 2 static three‐dimensional image sets with varying tumor sizes. Each of the 54 image sets contained both a CT and a CBCT scan.

### 2.2 4D image acquisition

This study employed a GE Discovery RT CT scanner combined with a Varian RGSC system to acquire 4D CT images by synchronizing CT scanning with real‐time respiratory motion tracking. A CIRS phantom with a predefined tumor motion trajectory was positioned on the CT scanner table, and simulated respiratory signals were recorded using the RGSC sensor, which integrated infrared optical tracking with real‐time signal processing. Axial cine scanning was carried out with the following parameters: a tube voltage of 120 kV, a tube current of 240 mA, a slice thickness of 1.25 mm, an acquisition interval of 10 mm, and a cine duration equal to 1.2 times the respiratory cycle.^16^ These parameters were representative of clinical 4D CT acquisition protocols for lung tumors at our institution. Following the scan, images were reconstructed using retrospective respiratory gating combined with a filtered back projection (FBP) algorithm, yielding a 4DCT dataset segmented into 10 phases.

Additionally, the VitalBeam linear accelerator was utilized to obtain 4D CBCT images of the phantom following the same predefined motion trajectory. The scanning parameters for this acquisition were as follows: an X‐ray voltage of 125 kV, 672 mAs, a frame rate of 7 fps, a total of 840 projections, and an imaging duration of 120 seconds. During scanning, the RGSC system sorted the projection data into phases based on the identical respiratory signals recorded from the phantom, and an advanced MKB algorithm was applied to reconstruct a 4D CBCT dataset comprising 10 phases.^17^

In this study, respiratory phases were binned into 10 equally spaced intervals (00–90) based on the external surrogate signal recorded by the RGSC system, where phase 00 corresponds to end‐exhalation and phase 50 corresponds to end‐inhalation, following the conventional amplitude‐based binning method. Phase alignment between 4DCT and 4DCBCT was ensured by using the identical respiratory signal from the phantom for both acquisitions, thereby synchronizing the phase assignment across modalities.

### 2.3 Image processing

To evaluate the accuracy of the registered contour, a rigid phantom tumor plug with known dimensions was utilized. Under static conditions, CT and CBCT images were acquired separately and imported into the PVmed automatic contouring system (PVmed, PV‐iCurve Pro 2.1). Using the lung window mode, multiple precise delineations of the tumor plug structure were performed, and the average of these results was designated as the edge threshold. This averaged threshold minimized inter‐observer variability and was consistently applied during the automatic contouring of all four‐dimensional images. The selection of this threshold was critical as it directly influenced the extracted tumor volume and, consequently, the DSC calculation; using a consistent threshold ensured comparability across all experimental conditions.

In Eclipse Image Registration Version 15.5, gray‐level based rigid registration was performed between the 4D CBCT (moving image) and the 4D CT (reference image). Focusing on quantitative reanalysis, the rigid registration method—known for its high reproducibility and minimal deformation errors—was employed. The image similarity metric employs mutual information (MI), which is suitable for multimodal registration, while the optimizer uses the gradient‐free Downhill Simplex algorithm. All registration processes are executed using a unified set of parameters to ensure consistent results. Fully automated registration was used to eliminate inter‐operator variability and ensure methodological consistency.

### 2.4 Evaluation metrics

According to the quantitative measurement method for image registration accuracy proposed in the AAPM TG132 report^18^ registration accuracy can be assessed by evaluating the degree of overlap between corresponding anatomical structures in the post‐registration reference and moving images. A commonly employed evaluation metric is the Dice similarity coefficient (DSC), which is defined as twice the overlapping volume of the two structures divided by the sum of their total volumes, that is, $\mathrm{DSC}=\frac{2|A\cap B|}{\left|A|+|B\right|}$. In this context, A represents the volume of the tumor plug‐in structure in the registered CT image, and B represents the volume of the tumor plug‐in structure in the registered CBCT image; the overlapping volume is calculated using Boolean operators within the Eclipse Image system. A DSC value approaching 1 indicates high consistency in the registered images, whereas a value of 0 signifies that the two structures do not overlap at all.

### 2.5 Data analysis

To systematically investigate the impact of respiratory cycle, tumor size, and motion amplitude on the registration accuracy of 4D CT‐4D CBCT images, this study employs two complementary data analysis methods. First, a random forest machine learning algorithm is applied to rank and quantify the importance of each variable^19^; subsequently, the statistical significance of the random forest results is validated using multifactor analysis of variance (ANOVA). All data analyses were conducted in a Python 3.12 environment using the scikit‐learn and statsmodels libraries.

Feature importance analysis based on random forest: We employed a random forest ensemble learning algorithm to assess feature importance.^20^ The model was initialized with 100 decision trees (*n*_estimators_ = 100), and the random state seed was fixed (random_state_ = 42). These were common default settings that ensure model stability and reproducibility. The dataset (54 samples) was partitioned into training and testing sets in an 80%–20% ratio (test_size_ = 0.2). The input features were respiratory cycle (*T*), tumor size, SI motion amplitude, AP motion amplitude, and LR motion amplitude. The dependent variables for separate models were the Dice similarity coefficient for average intensity projection (DSC_AIP_), Dice similarity coefficient for maximum intensity projection (DSC_MIP_), and each respiratory phase (DSC_00_–DSC_90_). Specifically, the DSC was calculated for the registered images at each of the ten respiratory phases individually. Model performance was evaluated using the root mean square error (RMSE) and the coefficient of determination (*R* ^2^). The RMSE was calculated as the square root of the average squared differences between predicted and observed values on the test set. The importance of each feature was extracted and visualized.

To further validate the statistical significance of the random forest analysis results, a multifactor ANOVA was employed. Specifically, an ordinary least squares (OLS) linear model was constructed using the AIP DSC and the MIP DSC as dependent variables, while the five experimental parameters served as independent variables. Type II ANOVA was then used to compute the sum of squares (sum_sq_), degrees of freedom (df), *F*‐statistic (*F*), and *p*‐value for each factor, with a significance level preset at *α* = 0.05.

## 3 RESULTS

### 3.1 Random forest feature importance analysis

**FIGURE 3:**
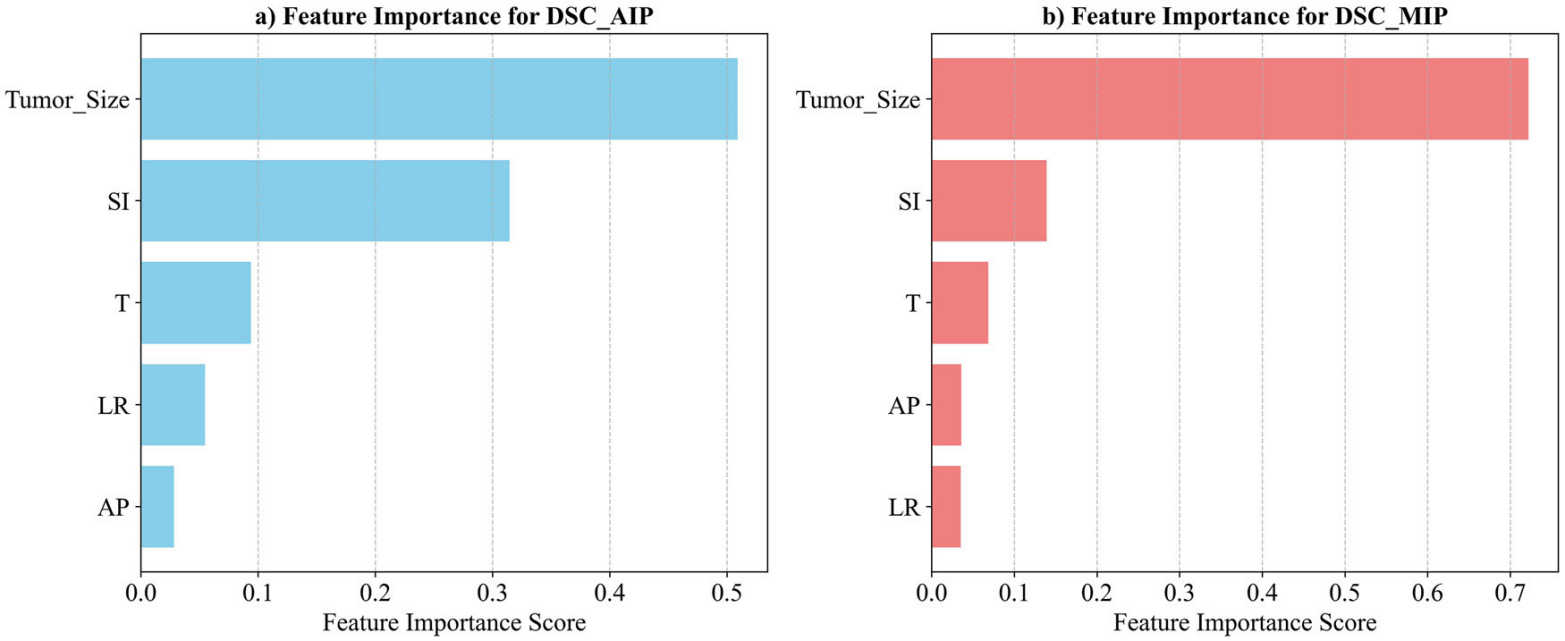
Results of AIP and MIP feature importance analysis. Bar plot showing the relative importance scores of respiratory cycle (T), tumor size, SI motion, AP motion, and LR motion for predicting the (a) average intensity projection (AIP) and (b) maximum intensity projection (MIP) Dice similarity coefficients. Note: The feature importance scores were calculated by the random forest model on the test set, and the sum of importance scores for all features is 1.

This study utilized a random forest regression model to systematically assess the impact of breathing cycle (T), tumor size, and motion along the SI, AP, and LR axes on the registration quality of AIP and MIP images in both 4DCT and 4DCBCT modalities. As illustrated in Figure 3, the feature importance analysis delineates the relative contributions of each factor to registration accuracy.

For the random forest model based on the average intensity projection (DSC_AIP_), performance metrics indicated a RMSE of 0.101 and an *R* ^2^ of 0.461. The factors were ranked by importance as follows: tumor size (importance score: 0.509) > SI motion (0.315) > breathing cycle (T) (0.094) > LR motion (0.055) > AP motion (0.028). Notably, tumor size emerged as the most influential factor, with an importance score nearly 1.6 times that of SI motion, the second most significant contributor.

Regarding the model in which the maximum intensity projection (DSC_MIP_) served as the basis, the performance metrics were more favorable, with an RMSE of 0.0321 and an *R* ^2^ of 0.8158. The ranking of factor importance exhibited a similar pattern with minor differences: tumor size (0.722) > SI motion (0.139) > breathing cycle (T) (0.069) > AP motion (0.036) > LR motion (0.035). It is particularly noteworthy that tumor size accounted for over 70% of the total importance in DSC_MIP_, thereby underscoring its decisive role in the registration process of MIP images.

**FIGURE 4:**
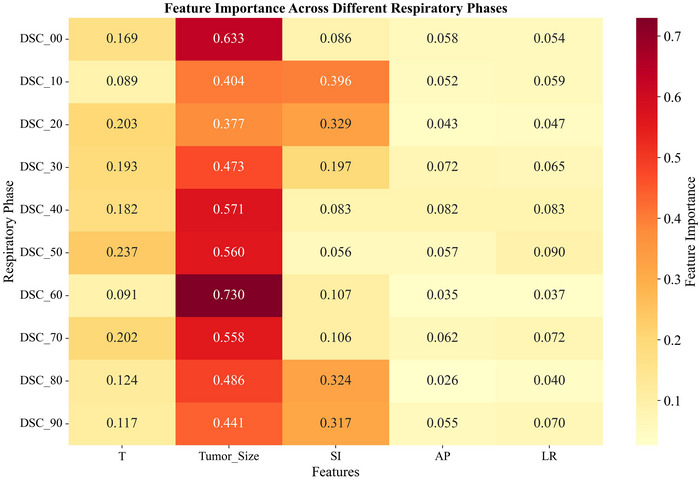
Feature importance across different respiratory phases. Line plot showing the importance scores of tumor size, SI motion, AP motion, LR motion, and respiratory cycle (T) for predicting the Dice similarity coefficient across ten respiratory phases (00 to 90). The RMSE for each phase is indicated above the plot. Note: The sum of feature importance scores for each respiratory phase is 1. The RMSE values shown are the test set errors of the model when predicting the DSC for that phase.

To further elucidate the characteristics of image registration across different respiratory phases, a phase‐by‐phase analysis (from DSC_00_ to DSC_90_) was conducted, with the corresponding results depicted in Figure 4 Across these phases, the RMSE values varied between 0.039 and 0.072, and the *R* ^2^ values ranged from 0.648 to 0.929. Tumor size dominates all respiratory phases (0.377–0.730). In DSC_10_, DSC_20_, DSC_80_, and DSC_90_, SI motion exceeds 0.3, showing increased sensitivity, while T, AP, and LR motions remain below 0.3 with minimal impact on registration quality.

### 3.2 ANOVA results

To evaluate the statistical significance of the random forest analysis results, a multifactor ANOVA was conducted. The results (see Table 1) indicate that, under the AIP condition, tumor size (*p* < 0.001) and motion in the SI direction (*p* < 0.001) significantly affect image registration quality, whereas respiratory cycle duration (T, *p* *=* 0.531) and motions in the AP (*p* *=* 0.114) and LR (*p* *=* 0.114) directions do not reach statistical significance. Under MIP, tumor size (*p* < 0.001) and SI direction motion (*p* *=* 0.002) also demonstrate highly significant effects, while the other factors remain statistically insignificant (*p* > 0.05). These ANOVA findings are consistent with the random forest feature importance analysis, jointly confirming that tumor size and SI direction motion are the primary factors affecting the registration quality between 4DCT and 4DCBCT images.

**TABLE 1:** Results of multifactor ANOVA.

	AIP				MIP			
Factor	sum_sq_	df	*F*	*p*	sum_sq_	df	*F*	*p*
Respiratory cycle, T	0.037	3	0.746	0.531	0.010	3	2.112	0.112
Tumor size	1.171	1	70.535	<0.001	0.235	1	153.457	< 0.001
SI	0.435	3	8.727	<0.001	0.026	3	5.757	0.002
AP	0.076	2	2.279	0.114	0.005	2	1.655	0.203
LR	0.076	2	2.279	0.114	0.005	2	1.655	0.203

*Note*: sum_sq_ = type II sum of squares; df = degrees of freedom (T:3, tumor size:1, SI:3, AP:2, LR:2); *F* = *F*‐statistic; *p* *=* *p*‐value.

### 3.3 Dice similarity coefficient distribution

**FIGURE 5:**
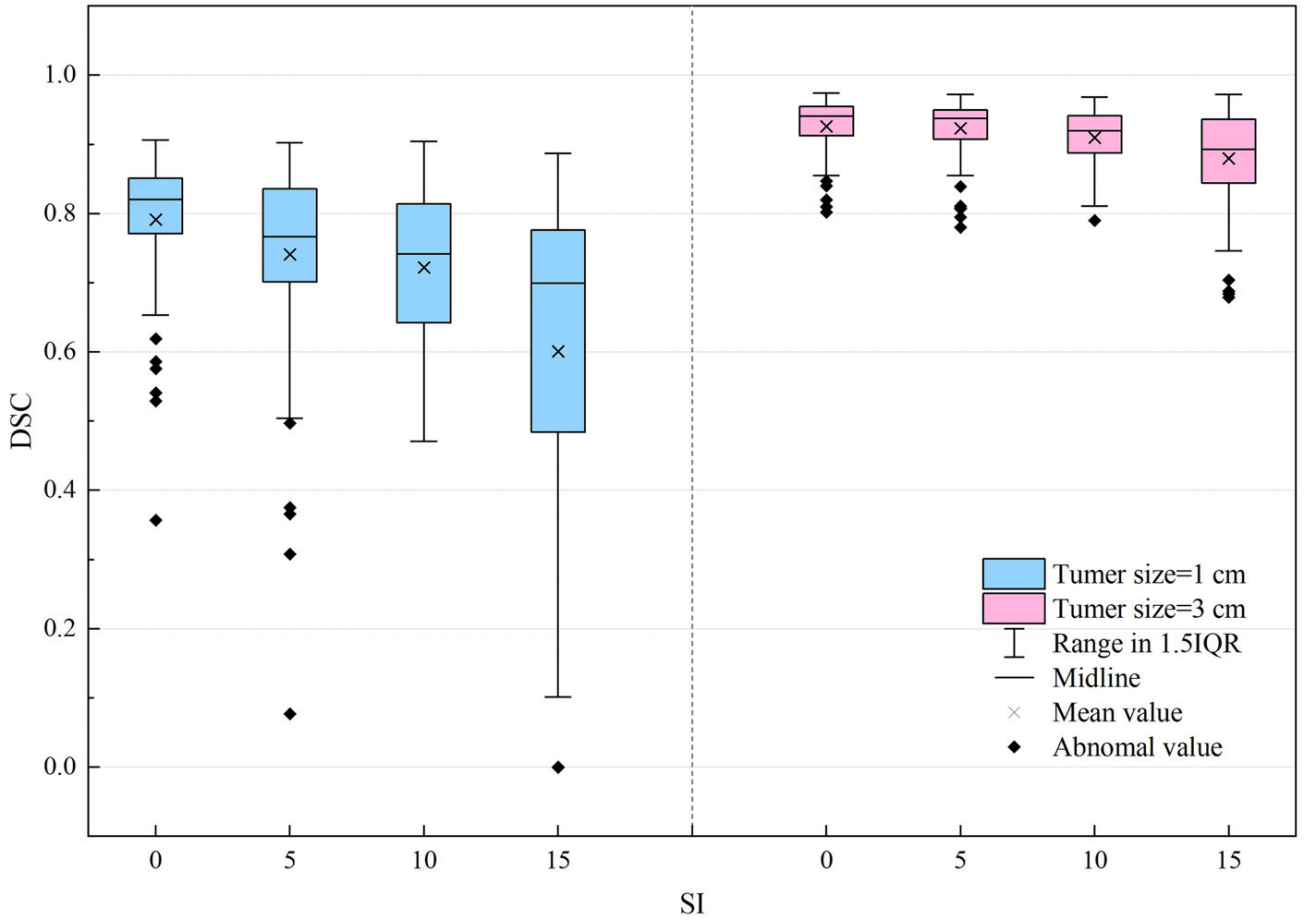
Distribution of Dice similarity coefficient values under various tumor sizes and SI‐direction motion amplitudes.

Figure 5 illustrates the distribution of Dice similarity coefficients as a function of tumor size and SI movement amplitude. Box plot analysis revealed several statistically significant characteristics.

First, tumor size has a considerable impact on the Dice similarity coefficient distribution. For 3 cm tumors, DSC values were generally high (with medians ranging from approximately 0.893–0.940) and exhibited a concentrated distribution, which indicates stable and reliable registration outcomes. In contrast, 1 cm tumors produced lower DSC values (with medians between approximately 0.699 and 0.821) and a noticeably broader distribution range, particularly when SI movement amplitude was large, suggesting greater variability in the registration results for small tumors.

Second, the SI movement amplitude was negatively correlated with the DSC value. As the SI movement amplitude increased from “low” (0 mm) to “high” (15 mm), the median DSC value gradually decreased, while the distribution range widened, indicating reduced registration accuracy with increasing movement amplitude. This effect was especially pronounced in 1 cm tumors, where the median DSC value in the high SI movement group was approximately 17% lower than that observed in the low movement group.

Notably, for 1 cm tumors with a 15 mm SI movement amplitude, the DSC value not only exhibited a significant decrease but also displayed markedly increased variability (IQR > 0.29). This observation suggests that this specific combination poses a considerable challenge for image registration and warrants particular attention in clinical practice.

## 4 DISCUSSION

This study combined random forest analysis with ANOVA to systematically evaluate the impact of multiple factors on the registration quality between 4D CT and 4D CBCT images. The results consistently demonstrated that tumor size and motion in the SI direction are the two most critical factors influencing registration accuracy, whereas the respiratory cycle and motions in the AP and LR directions exhibited comparatively minor effects. While the influence of tumor size and major respiratory motion was recognized clinically, this study provides a quantitative ranking of their relative importance and confirms their statistical dominance over other factors through a controlled, multi‐factorial experiment. These findings are consistent with and extend previous literature on the challenges of 4D image registration^7^, ^21^ particularly highlighting the vulnerability of small tumors under large respiratory motion using a comprehensive multi‐factor analysis approach.

The prominence of tumor size as the most influential factor aligns with clinical experience. Larger tumors (3 cm) are associated with higher and more stable Dice similarity coefficients, owing to their greater volume, clearer boundaries, and improved image contrast, which facilitate more accurate identification and registration. In contrast, small tumors (1 cm) present considerable challenges due to partial volume effects and reduced contrast, leading to greater variability in registration outcomes. This underscores the necessity for refined registration strategies—such as optimized imaging protocols, advanced registration algorithms, or multimodal image fusion—in cases involving small tumors.

The significant impact of SI‐direction motion highlights the particular challenges posed by respiratory motion in 4D image registration. The SI direction is the principal axis of diaphragmatic motion and lung expansion, typically exhibiting the largest amplitude, which can lead to substantial deformation and complex changes in image features, including motion blurring in both 4DCT and 4DCBCT. Our phase‐specific analysis further revealed that the influence of SI motion varies across respiratory phases, with increased importance during end‐inspiration and end‐expiration phases (e.g., DSC_10_, DSC_20_, DSC_80_, DSC_90_). This phase‐dependent sensitivity reflects the nonlinear nature of respiratory motion and suggests potential for developing phase‐adaptive registration algorithms that tailor strategies according to respiratory phase characteristics.

The relatively limited impact of the respiratory cycle (*T*) may indicate that current 4D imaging and registration techniques are relatively robust to variations in breathing period. Modern systems often use external surrogate signals for respiratory phase binning, and registration algorithms may effectively compensate for cycle length variations. Nevertheless, extreme respiratory patterns (e.g., very short or very long cycles) or the use of non‐corresponding respiratory signals between 4D CT and 4D CBCT acquisition^22^ were not fully explored in this study and may warrant further investigation to assess registration robustness under more realistic, inconsistent breathing conditions.

The minimal effects observed for AP and LR motions are likely attributable to their smaller physiological amplitudes—the maximum amplitude set in our experiment was only 5 mm, which may not fully represent clinical scenarios where larger motions occasionally occur. In certain clinical situations, such as lateral decubitus positioning or tumors in specific anatomical regions, these motions may become more relevant. Thus, while our findings suggest a lower overall impact, context‐specific evaluation remains important.

The superior performance of the random forest model for MIP‐based registration (higher *R* ^2^) compared to AIP may be attributed to the inherent properties of MIP images. MIP preserves the highest intensity along projection lines, which can enhance the visibility of high‐contrast structures like tumor boundaries throughout the motion cycle, potentially providing more consistent features for the registration algorithm and the predictive model.

### 4.1 Clinical implications

Our results carry important practical implications for the clinical implementation of 4D CBCT, particularly in workflows involving plan adaptation, motion assessment, and quality assurance (QA):

1. For patients with small tumors (e.g., ≤1 cm in diameter) and large SI motion (e.g., ≥10 mm), additional motion management strategies—such as respiratory gating, breath‐hold techniques, or real‐time tracking—should be considered to enhance registration accuracy before proceeding with adaptive planning based on 4D CBCT.
2. SI motion amplitude could serve as a key QA metric in clinical evaluations of image registration. Patients exhibiting large SI motion, especially combined with small tumor volume, may require more frequent or meticulous registration checks.
3. In resource‐limited settings, prioritizing registration protocol optimization for small tumors with significant SI motion may yield the greatest clinical benefit.
4. The potential use of 4D CBCT for geometric verification and motion evaluation is supported, but its application for direct dosimetric calculation in adaptive radiotherapy for small, mobile targets requires caution and further validation.

### 4.2 Limitations and future directions

This study has several limitations. First, the use of a motion phantom, while ensuring controllability and reproducibility, may not fully replicate the complexity of patient breathing patterns, anatomical variability, or the scenario of non‐synchronized respiratory signals between CT and CBCT acquisitions. Second, the evaluation relied primarily on the Dice similarity coefficient; incorporating additional geometric accuracy metrics (e.g., Hausdorff distance, target registration error) could provide a more comprehensive assessment. Third, the motion amplitudes in AP and LR directions were limited to 5 mm, which may not cover all clinical cases. Future studies should validate these findings in patient cohorts, explore the performance of advanced registration methods (such as deep learning‐based or deformable registration techniques) under challenging conditions involving small tumors and large respiratory motions, and investigate the impact of respiratory signal inconsistency on registration robustness.

## 5 CONCLUSION

This study employed machine learning and statistical methods to identify the key factors affecting the registration quality between 4D CT and 4D CBCT images. The main conclusions were as follows: Tumor size was found to significantly influence registration outcomes, with the registration accuracy for a 3 cm tumor being markedly superior and more stable than that for a 1 cm tumor. This indicated that aligning small tumors posed greater challenges, necessitating increased clinical attention. SI direction motion was also shown to affect registration quality; an increase in motion amplitude markedly reduced registration accuracy and increased surface distance errors, particularly in cases involving small tumors. Consequently, close clinical monitoring was advised. These findings provided quantitative guidance for optimizing 4D image registration strategies in clinical IGRT workflows, especially for challenging cases involving small, mobile tumors.

## AUTHOR CONTRIBUTIONS

Qiaoyan Jing: conceptualization, methodology, investigation, data curation, formal analysis, writing—original draft. Shuyu Lin: software, validation, visualization. Binyun Huang, Tingjun Luo, Xianya Li: resources, investigation. Weiming Zhang: supervision, project administration. Shaohan Sun: conceptualization, resources, supervision, writing—review & editing, funding acquisition. All authors reviewed and approved the final manuscript.

## FUNDING

This research was supported by Youth Science Foundation of Guangxi Medical University (grant number: GXMUYSF202450). This study also received support from the Guangxi Medical and Health Appropriate Technology Development (S2023097) and Promotion Application Project and the Guangxi Medical and Health Self Funded Project (Z20201311).

## CONFLICT OF INTEREST STATEMENT

The authors declare no conflicts of interest.
